# Intraocular Suture Looping Technique for Flapless Four-Point Refixation of Dislocated Intraocular Lenses

**DOI:** 10.1155/2021/6648777

**Published:** 2021-02-08

**Authors:** Xin Hu, Bo Zhao, Haiying Jin

**Affiliations:** ^1^Department of Ophthalmology, Huaihe Hospital, Henan University, Kaifeng 475000, China; ^2^Department of Ophthalmology, Tenth People's Hospital Affiliated to Shanghai Tongji University School of Medicine, Shanghai 200072, China

## Abstract

**Purpose:**

To describe a flapless/grooveless technique for four-point refixation of a dislocated intraocular lens (IOL) with four fenestrated haptics.

**Methods:**

An intraocular suture looping technique was performed with the assistance of two 27-gauge needles. A looping needle was passed into the eye through paracentesis and was used to loop both fenestrated haptics on the same side with an 8–0 polypropylene thread. A guiding needle was used to guide the looping needle out of the eye at the scleral fixation sites. After looping each pair of fenestrated haptics on nasal/temporal sides with 8–0 polypropylene sutures, the IOL was refixated by definitive knotting. The exterior suture ends were buried into the sclera without the creation of scleral flaps/grooves.

**Results:**

The technique was employed in four eyes (three patients). The mean postoperative follow-up period was 13.8 ± 2.2 months. Postoperatively, the IOLs of all the eyes remained well positioned and stable. The postoperative visual acuities of all the eyes were improved. No suture erosion, hypotony, scleral atrophy, chronic inflammation, retinal tears, and/or detachments were observed within the follow-up period.

**Conclusion:**

The present technique provides minimal surgical invasion for the transscleral refixation of a dislocated IOL with four fenestrated haptics.

## 1. Background

There are various options for managing a dislocated intraocular lens (IOL) according to the status of dislocation and the type of IOL implanted [1–5]. A dislocated IOL-capsular bag complex or IOLs with closed-loop haptics can be managed by looping the encapsulated haptic or eyelet of the haptic with a suture for fixation [6–9]. Cases of completely dislocated IOLs with four fenestrated haptics present a unique challenge. To the best of our knowledge, no reports have been published on the performance of four-point intraocular refixation of IOLs with four fenestrated haptics. Transscleral four-point suture fixation for secondary IOL implantation without sufficient capsular support, performed by looping each pair of fenestrated haptics, provides several advantages, including avoidance of IOL tilt and postoperative pupil capture, enhanced IOL stability and centration, and a low risk of pigmentary dispersion glaucoma and cystoid macular edema [10–15]. Suture looping manipulation through two pairs of fenestrated haptics performed outside the eye for a secondary implantation is relatively straightforward. However, for a dislocated IOL with four fenestrated haptics, a four-point refixation requiring looping the pairs of fenestrated haptics inside the eye is technically difficult. Recently, we have presented a method of refixating an IOL with closed-loop haptics, namely, the “intraocular looping technique,” to pass a suture through the closed-loop of the IOL haptic in the eye [16]. The technique can be further modified for four-point refixation of a special type of IOL. It provides minimal surgical invasion without the creation of scleral flaps, pockets, or grooves. It can also be performed transconjunctivally without conjunctival dissections.

## 2. Methods

A retrospective analysis of patients who underwent flapless four-point refixation of dislocated IOLs (Akreos AO60, Bausch and Lomb, North Clearwater, FL) with four fenestrated haptics enrolled between January 2019 and July 2020 was performed. The present study adhered to the tenets of the Declaration of Helsinki and was approved by Institutional Ethics Board of Tenth People's Hospital affiliated to Shanghai Tongji University School of Medicine. Written informed consent was obtained from all patients. All patients provided informed consent after description of the nature and consequences of the study. Data collection included demographic details, indication for surgery, intraoperative and postoperative complications, follow-up duration, preoperative and postoperative intraocular pressure, visual acuity, IOL position evaluated by anterior segmental photograph and the Scheimpflug imaging system (Pentacam, Oculus Optikgeräte GmbH, Wetzlar, Germany), posterior segment photograph, and optical coherence tomography (RTVue-100, Optovue Inc., Fremont, CA, US) for macular evaluation.

### 2.1. Surgical Technique

**Figure 1:**
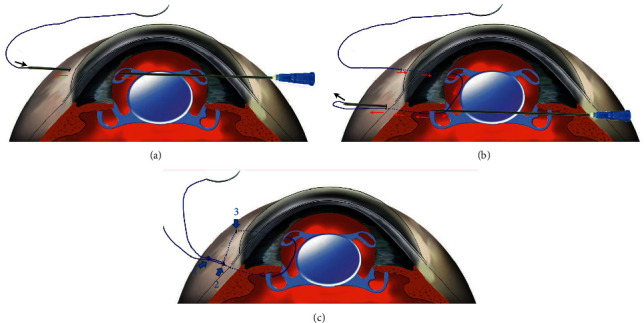
Schematic figure of the intraocular looping technique for four-point fixation (drawn by Haiying Jin). This figure is authorized to be published by the present journal to demonstrate the surgical procedures. (a) The looping needle is externalized from the first fixation point and loaded with an 8–0 polypropylene thread in its lumen (arrow). Red arrows show the needle path from the eyelet of the IOL to the sclerotomy. (b) After loading the thread, the looping needle is recoiled into the anterior chamber and passed through the following paths: the first eyelet of the IOL, the second eyelet of the IOL, and ab externo pass through the second sclerotomy (red arrows). The thread in the lumen of the needle is finally pulled out (black arrow). A suture loop is created passing through both eyelets on the same side. (c) After the intrascleral passing (arrow 3) of the thread from the first fixation site to the second fixation site, a double knot technique is performed for a flapless IOL fixation. The first overhand knot in the sclerotomy (arrow 2) is used for the IOL fixation. The second knot (1) is used to lead the ends of the threads for burying in the sclera.

All the surgeries were performed under retrobulbar anesthesia by one of the authors (H.J.). The supplemental video (Video, Supplement Digital Content 1) and Figure 1 demonstrate the procedures. For cases with a completely dislocated IOL-capsular bag complex dropping into the vitreous cavity, complete 25-gauge pars plana vitrectomies were performed. The two pars plana sclerotomies were placed 2 mm from the limbus and were designed as the two superior sites for IOL fixation. The IOL was gripped with end-griping forceps and placed above the iris plane. The IOL was orientated horizontally. The four-point fixation sites were placed superiorly and inferiorly to 3 and 9 o'clock, 2 mm posterior to the limbus, and 5 mm apart. Two corneal paracenteses were separately performed, approximately 180 degrees apart on the nasal and temporal sides. Two 27-gauge needles (or 27-gauge/30-gauge needles) were bent at the hubs. An ab externo penetration was performed with one needle (the guiding needle) at the inferior fixation site 2 mm from the limbus on the temporal side. The other needle (the looping needle) was passed into the eye through the previous created corneal paracentesis located on the opposite side. The intraocular suture looping technique modified from our previously published method was performed in a bimanual manner. [15]. Either of the two needles was passed through the eyelet of the inferior haptic. The tip of the looping needle was then docked into the guiding needle in a needle-needle manner and was guided out of the eye. An 8–0 polypropylene thread (Prolene, Polypropylene Suture; Ethicon, Johnson-Johnson, New Brunswick, NJ) was bisected. The end of one half of the suture was inserted into the lumen of the looping needle for approximately 4-5 mm. The other side of the thread connected to a curved needle was left outside the eye. The looping needle with the thread was then recoiled into the globe. An ab externo passage of the guiding needle was then performed through the superior fixation site. The same method was performed to pass the looping needle through the eyelet of the superior haptic. The looping needle in the anterior chamber was guided out of the superior fixation site in a similar manner. The end of the suture in the looping needle was pulled out from the needle lumen with forceps. A suture loop was thus created passing through the pair of fenestrated haptics on the temporal side. The same set of manipulations was performed to loop the pairs of fenestrated haptics on the nasal side. Thus, two pairs of suture loops for a four-point IOL fixation were created. The curved needle attached to the external suture was started with an intrascleral pass from the inferior fixation site to the adjacent transscleral penetration site parallel to the limbus. A second intrascleral pass of the needle from the exiting site of the sclera to the superior fixation site was performed. The same set of manipulations was performed for the nasal side. After adjusting the suture tension on both sides to center the IOL, the two ends of the suture were tied for definitive knotting fixation (the fixation knot) into the sclerotomy for both sides. Another overhand knot (anchor knot) was then created approximately 3 mm from the first knot. The technique of burying the anchor knot and the ends of the sutures into the scleral tunnel was identical to our previous publication [15, 16]. A 27-gauge needle was used to create an intrascleral tunnel from the sclerotomy approximately 3-4 mm in length parallel to the limbus aiming either superiorly or inferiorly. The curved needle, connected to the anchor knot, was introduced with an intrascleral pass from the sclerotomy to the adjacent transscleral penetration site through the scleral tunnel. After pulling out the needle transconjunctivally, the attached suture was further pulled to bury the second knot and the suture ends in the sclera. The externalized ends of the sutures were cut flush to the scleral surface. The conjunctival openings were left sutureless or closed with a one-stitch 10–0 nylon suture.

## 3. Results

**Figure 2:**
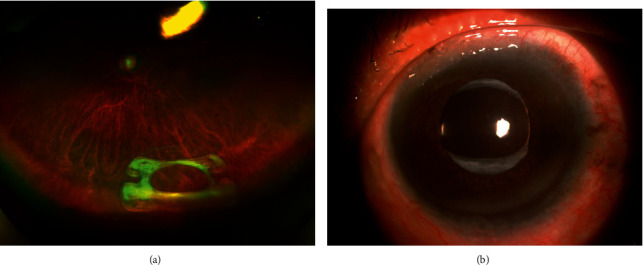
(a) Preoperative overview of a dislocated IOL-capsular bag complex (arrow) in the vitreous cavity. (b) Postoperative overview of the well-positioned four-point refixated IOL-capsular bag complex. No erosion or exposure of the trimmed ends of the sutures and no scaring of the conjunctiva were observed.

The technique was adopted in four eyes of three patients (2 male and 1 female) with a mean age of 56 ± 8 years. The mean follow-up period was 13.8 ± 2.2 months (range 11–16 months). Uncorrected visual acuities improved from a mean of 1.10 ± 0.08 logMAR (Snellen 20/250) preoperatively to 0.22 ± 0.05 logMAR (Snellen 20/32) at the final follow-up. No intraoperative surgical complications were observed. The IOLs remained well centered throughout the follow-up period. No erosion or exposure of the trimmed ends of the sutures was observed (Figure 2). No postoperative complications of hypotony, elevated intraocular pressure, hyphemia, vitreous hemorrhage, abnormal inflammation, cystoid macular edema, or retinal detachment were observed during the postoperative follow-up period.

## 4. Discussion

Several strategies have been published for the management of dislocated IOLs or an IOL-capsular bag complex, including IOL exchange and refixation of the dislocated IOL using various methods [1–5]. Refixation techniques are less surgically traumatic due to the avoidance of creating a large corneal/scleral incision for the IOL exchange. To the best of our knowledge, no publications have been reported on a four-point refixation of a dislocated IOL typed with four fenestrated haptics. We have previously presented an intraocular looping technique to loop the suture through the fenestrated haptics for two-point IOL refixation in our previous publication [16]. The technique involves manipulations with two needles, a guiding needle and a looping needle, and has multiple advantages, including simplified surgical procedure, satisfactory maneuverability, and avoidance of haptic-externalization or repeated passing of long needles through the globe. In this study, the intraocular looping manipulation was further modified to loop a pair of fenestrated haptics for four-point fixation other than a single haptic. After loading the suture into the looping needle, the needle was passed through the eyelet of the first haptic, sequentially passed through the eyelet of the second haptic, and finally passed out from the second fixation site to deliver the suture end for its externalization.

Another approach adopted in this technique for minimizing surgical trauma is intrascleral manipulation, similar to our published techniques [15, 17, 18]. The suture was passed intrasclerally from the fist fixation site to the second one with the aid of an attached curved needle. Fixation of the IOL was accomplished by tying the two ends of the suture in the sclerotomy. As the tensions of the bilateral suture loops were adjusted before fastening the knots, well centration of the IOL can be achieved. The second knot, approximately 3 mm from the fixation knot, was used for intrascleral anchoring of the suture ends without creating scleral flaps, pockets, or grooves. The manipulations can be performed under sutureless small conjunctival incisions and, therefore, greatly reduce the surgical trauma that requires the creation of large conjunctival incisions and scleral flaps. As the suture cutting ends lie tangential to the sclera, the technique inherits the major advantage of the friction knot method for preventing suture erosion. [19]

A limitation of this study is its small sample size due to relatively rare occurrence of dislocation of the type of IOL with four fenestrated haptics. Studies evaluating more cases might be necessary to evaluate other potential complications.

In conclusion, this technique represents a safe, effective, minimally invasive procedure for the management of dislocated IOL/IOL-capsular bag complex with four fenestrated haptics.

## Data Availability

The datasets analyzed during the current study are available from the corresponding author upon request.

## Supplementary Materials

Supplementary Materials Supplemental digital content: Video1. Intraocular suture looping technique for flapless four-point refixation of dislocated intraocular lenses.
